# IRF4 and STAT3 activities are associated with the imbalanced differentiation of T-cells in responses to inhalable particulate matters

**DOI:** 10.1186/s12931-020-01368-2

**Published:** 2020-05-24

**Authors:** Jinzhun Wu, Dandan Ge, Taoling Zhong, Zuojia Chen, Ying Zhou, Lingyun Hou, Xiaoliang Lin, Jiaxu Hong, Kuai Liu, Hui Qi, Chaoying Wang, Yulin Zhou, Cheng Li, Chuan Wu, Shuiping Wu, Zuguo Liu, Qiyuan Li

**Affiliations:** 1 Department of Pediatrics, the First Affiliated Hospital of Xiamen University, Xiamen, 361003 China; 2 Maternal and Child Health Hospital Affiliated Xiamen University, Xiamen, 361102 China; 3 Pediatric Key Laboratory of Xiamen, Xiamen, 361003 China; 4 Medical Research Institute of Children, School of Medicine, Xiamen University, the First Affiliated Hospital of Xiamen University, Xiamen, 361003 China; 5 National Institute of Data Science in Health and Medicine, School of Medicine, Xiamen University, Xiamen, 361102 China; 6 Experimental Immunology Branch, National Cancer Institute, NIH, Bethesda, MD USA; 7 Department of Ophthalmology and Vision Science, Eye & ENT Hospital, Fudan University, Shanghai, 200032 China; 8 Shanghai Key Laboratory of Visual Impairment and Restoration, Shanghai, 200032 China; 9 NHC Key Laboratory of Myopia, Fudan University, Shanghai, 200032 China; 10 The 980st Hospital of the PLA Joint Logistics Support Force (Primary Bethune International Peace Hospital of PLA), Shijiazhuang, Hebei 050082 China; 11 Fujian Provincial Key Laboratory of Ophthalmology and Visual Science, Eye Institute of Xiamen University, School of Medicine, Xiamen University, Xiamen, 361102 China; 12 Marine Chemistry and Toxicology Research Center, College of the Environment & Ecology, Xiamen University, Xiamen, 361102 China

**Keywords:** Particulate matters, Gene expression profiling, Allergic respiratory disease, Imbalanced T-cell differentiation, Transcription factor

## Abstract

**Background:**

Particulate Matter (PM) is known to cause inflammatory responses in human. Although prior studies verified the immunogenicity of PM in cell lines and animal models, the effectors of PM exposure in the respiratory system and the regulators of the immunogenicity of PM is not fully elucidated.

**Methods:**

To identify the potential effector of PM exposure in human respiratory system and to better understand the biology of the immunogenicity of PM, We performed gene-expression profiling of peripheral blood mononuclear cells from 171 heathy subjects in northern China to identify co-expressed gene modules associated with PM exposure. We inferred transcription factors regulating the co-expression and validated the association to T-cell differentiation in both primary T-cells and mice treated with PM.

**Results:**

We report two transcription factors, IRF4 and STAT3, as regulators of the gene expression in response to PM exposure in human. We confirmed that the activation of IRF4 and STAT3 by PM is strongly associated with imbalanced differentiation of T-cells in the respiratory tracts in a time-sensitive manner in mouse. We also verified the consequential inflammatory responses of the PM exposure. Moreover, we show that the protein levels of phosphorylated IRF4 and STAT3 increase with PM exposure.

**Conclusions:**

Our study suggests the regulatory activities of IRF4 and STAT3 are associated with the Th17-mediated inflammatory responses to PM exposure in the respiratory tracts, which informs the biological background of the immunogenicity of particulate matters.

## Introduction

Particulate Matter (PM) is a major cause of air pollution and a risk factor to public health. In 2015, 4.241 million deaths worldwide are attributable to ambient particulate matter [1], which is ranked the 6th of the 10 largest hazard factors contributing to the global disability-adjusted life-years (DALYs) [1]. Epidemiological studies conducted in different regions of the world confirm that PM is associated with elevated incidences of various human diseases, including cardiovascular disease [2, 3], lung cancer [4, 5], chronic obstructive pulmonary disease (COPD) [6], Atopic dermatitis (AD) [7, 8], hypertension [9], type 2 diabetes [10], dry eye [11], allergic rhinitis [12] and asthma [13].

PM is classified into three subtypes, PM_2.5_, PM_10_ and ultra-fine particles (UFP), according to the aerodynamic diameters. The physical-chemical properties of PM differ according to the geological locations, the subtypes and the sources. Most of the natural PM particles consist of mineral dusts, metal and ammonium salts. Anthropogenic PM, however, consists mainly particles of black carbon, various organic and inorganic residues of combustion of fossil fuels or coals. PMs are generated from oxidation of primary airborne pollutants such as sulfur and nitrogen oxides, which is also known as secondary particles [14]. Moreover, PM absorbs other airborne molecules such as heavy metals, polycyclic aromatic hydrocarbons (PAHs) in the surface.

Exposure to PM are proved to be toxic to humans. Although the biological background of the toxicity of PM is not fully elucidated, most evidences suggest several biological processes are involved in the cells’ responses to PM. First, PM cause various immune responses in human tissues. In vitro and in vivo analyses suggest that PM induces neutrophils, eosinophils as well as macrophages by activating pro-inflammatory cytokines, such as IL8, IL1β and GM-CSF, which trigger a series of inflammatory responses. In addition, PM activates Th2-related cytokines (IL-33, ST2) while suppresses Th1-related cytokines (INF-γ), which leads to the imbalance among T-helper cells. Two transcription factors, GATA3 and T-bet are shown to be associated with the responses to the exposure of PM in mice but the observations are not statistically significant [15]. Moreover, exposure to PM also causes oxidative stress, which is mediated by Nrf2 in the PIK3/AKT signaling pathway [16]. Plus, PM also induces apoptosis and autophagy via TNF-α and caspase signaling [17].

Respiratory tracts, which interact directly with inhalable PM, are the major site of PM deposition in human body. Many prior evidences claim that the exposure is associated with allergies in the respiratory tracts, such as chronic obstructive pulmonary disease (COPD) [6], allergic rhinitis [12] and asthma [13]. However, most of the evidences are based on cell lines and mice; and less is known about how cells react to PM exposure in human.

To fully assess the effects of PM on human respiratory tracts, we generated a peripheral blood gene-expression profiles of a healthy population under PM exposure; we developed an integrated method to infer specific transcription factors that modulate T-cell response to the PMs and verified the effects of PM on T-cell differentiation in vitro and in vivo. Our study reveals the biological background of the immunogenicity of PM and helps to understand the toxicity of PM in allergic respiratory disease.

## Materials and methods

### Study cohort

The subjects are randomly chosen from healthy individuals who were enrolled in physical examinations in three cities in northern China, Beijing, Taiyuan and Shijiazhuang. All subjects are from Chinese population without recorded underlying diseases. The collection and usage of the patients’ peripheral blood sample has received written consent. This study protocol and informed consent form were overseen and approved by a steering committee, institutional review boards of Xiamen University and the First Affiliated Hospital of Xiamen University. All methods were carried out in accordance with the relevant guidelines and regulations.

The air quality record of the three cities during the study period, including daily concentration of CO, SO_2_, NO_x_, PM_10_ and PM_2.5_, is available from the Ministry of Environmental Protection of the People’s Republic of China (www.mee.gov.cn).

### Gene expression profiling

Total RNA was isolated from human peripheral blood mononuclear cells (PBMCs) and hybridized to the Illumina single color Human BeadChip HT12 v4 whole genome expression array. The gene expression profiles were analyzed using the “limma” package available in R-3.5. Genes with significant detecting power (*P* < 0.05) were selected for further statistical analyses.

### Statistical analysis

#### Deriving co-expressed gene modules

We used weighted gene co-expression network analysis (WGCNA) to generate a co-expression subnetwork of genes from the expression profiles [18]. Then we calculated the Spearman’s rank correlation coefficients between the module eigengenes and the concentrations of PM_2.5_, PM_10_ and other pollutants such as CO, SO_2_ and NO_x_. We then used “TFactS” to predict transcription factors of which the activity is significantly enriched in each co-expression modules (FDR < 0.1) [19].

#### Motif analysis

For each gene in a co-expressed module, we retrieved the genomic DNA sequences corresponding to the DNaseI hypersensitivity sites (DHS) located within 5 kilobases of either side of the transcription starting sites (TSS). The location of DNase I hypersensitivity sites are based on Digital DNaseI Hypersensitivity Clusters from ENCODE [20]. Then we used HOMER (version 4.9) to evaluate the enrichment of known transcription-factor-binding motifs [21]. As the activities of the transcription factors are tissue specific, we only keep significantly enriched motifs (FDR < 0.1) in relevant T-cell types.

### Particles

Ambient PM (aerodynamic diameter ≤ 10 μm) were collected in an urban area of Xiamen, China using a high-volume ultrafine particle sampler with a Zefluor filter. After collection, PM was dehydrated and dispersed the PM in 2 mg/ml Sodium carboxymethylcellulose (CMC) ultrasonically. A total of eight water soluble ions (Cl^−^, NO3^−^, SO42^−^, Na^+^, NH4^+^, K^+^, Mg^2+^, and Ca^2+^) were analyzed by ion chromatography. A portion of filter sample was digested for element measurement (K, Ca, Mg, Al, Fe, Be, V, Cr, Mn, Ni, Cu, Zn, As, Se, Cd, Ba, and Pb) using inductivity coupled plasma-mass spectrometry (ICP-MS) (7700X, Agilent). An area of 0.49 cm^2^ punched from each quartz filter was analyzed for organic carbon (OC) and elemental carbon (EC) fractions using a DRI model 2001 carbon analyzer (Atmoslytic, Calabasas, CA).

To assess the proportion of PM_2.5_ and PM_10_ in our sample, we studied the distribution of the diameters of the particulates in the PM sample used for the in vivo experiments by microscope observation.

In addition, the protease activity of PM was measured by Protease Fluorescent Detection Kit (PF0100, Sigma) following protocols described by the manufacturer. We used the 50 ng and 30 ng Trypsin Control as the standard sample, and the loading concentration of PM sample is 10 mg/ml. A reading equal to 120% of the value obtained with the blank sample (0 ng trypsin) is considered significant.

### In vitro analysis

Single naïve CD4^+^ T-cells were isolated from the mouse (BALB/c) spleen aged 8–10 weeks and purified by CD4 (L3T4) MicroBeads. Isolated CD4^+^ T-cells were stimulated with 5 μg of plate-bound anti-CD3 and 1 μg/ml of soluble anti-CD28.After co-cultivation with PM for 48 h, T-cell differentiation was analyzed using BD LSRFortessa™ flow cytometer following standard protocols (Additional file 1).

### Animal model

Wild type BALB/c mice (female, 6–8 weeks) were obtained from the Xiamen University Laboratory Animal Center (XMULAC). All mice were maintained under specific pathogen-free (SPF) condition. The animal experiment was approved by the Institutional Animal Care and Use Committee (Laboratory animal license: SYXK (Min) 2018–0010) and was in accordance with good animal practice as defined by the XMULAC (Xiamen Univerisity Laboratory Animal Center).

Mice (BALB/c) were randomly divided into two groups. The treatment group was given 40 μl PM suspension (containing 100 μg PM) intranasally for 3 consecutive days, and the control group was given 40 μl NaCl solution. All the mice were anesthetized through nose with isoflurane before dripping.

#### Flow Cytometry analysis

We sacrificed the mice at 18 h, 24 h, 40 h, and 72 h after the last nasal drip. Then we harvested the lung tissue and collected the lymphocytes for flow cytometry analysis (Additional file 1).

#### ELISA, quantitative real-time PCR and Western blotting

We sacrificed the mice at 24 h after the last nasal drip. To measure cytokines expression, we performed capture ELISA for IL-17A, IL-21, IL-22, IL-4 and IL-13 in supernatants of primary T-cells, blood and bronchoalveolar lavage fluid (BALF) from mice treated with PM suspension by Meso-Scale Discovery (MSD) platform (K151VBK-1, sensitivity < 0.6 pg/ml), univ-bio, Shanghai, China.

To measure the transcript levels of relevant genes (Irf4, Batf, Stat3, IL-4), we performed quantitative real-time PCR was performed using SYBR green-based reagents on the ViiA 7 Real-Time PCR System (Life Technologies) with primer pairs targeting the cDNAs of interests (see Additional file 1: Table S1). All qPCR reactions were run in duplicates and the resulted CT values were normalized to β-actin (Additional file 1).

Phosphorylated and unphosphorylated STAT3 and IRF4 in mouse lung were separated by SDS-polyacrylamide gel electrophoresis then western blotting was conducted using IRF4 (D9P5H) Rabbit mAb, phospho-STAT3 (Tyr705) (M9C6) Mouse mAb, STAT3 (124H6) Mouse mAb and phospho-IRF4 (Phospho-Tyr122/125) antibody.

## Results

### Co-expressed gene modules correlated with PM exposure

**Fig. 1:**
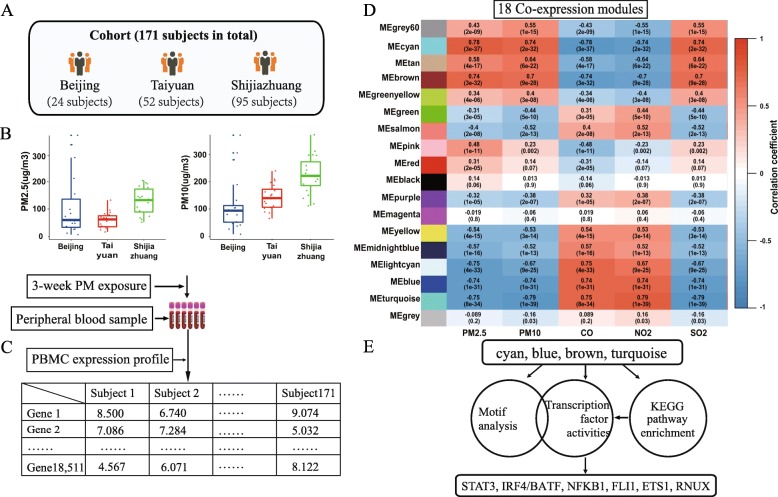
Peripheral blood mononuclear cell (PBMC) expression profiling in a healthy population reveals co-expressed gene modules in association with PM exposure. **a**, enrollment of healthy subjects from three cities in north China, Beijing, Taiyuan and Shijiazhuang; **b**, Three-week average exposure level (μg/m^3^) of PM_2.5_ (left) and PM_10_ (right) of each enrolled subject from the three cities; **c**, PBMC gene expression profile generated from the enrolled subjects; **d**, 18 co-expressed gene modules retrieved from the PBMC gene expression profiles with correlation coefficients to the exposure levels of five major pollutants shown heat colors and **e**, integrating DNA motif, transcription factor activity and KEGG pathway to infer transcription factors underlying the co-expressed gene modules (* *P* < 0.05, ** *P* < 0.01, *** *P* < 0.001)

We enrolled 171 healthy subjects from three cities in northern China, namely, Beijing, Taiyuan and Shijiazhuang (Fig. 1a). These cities are highly homogeneous in terms of the geographic features and climatic conditions. To measure the exposure to the PM and other pollutants, we calculated the average concentrations of five pollutants (PM_2.5_, PM_10_, CO, NO_2_, SO_2_) within 21 days preceding the collection of the blood sample in each city (Fig. 1b, see Additional file 1: Figure S1, and Table 1). According to the U.S. Environmental Protection Agency (EPA) [22], 2012, the nation’s air quality standards for PM_2.5_ is 35 μg/m^3^. The concentration of PM_2.5_ of the three cities during the study period ranged from 13.20 μg/m^3^ to 4315.64 μg/m^3^, which was classified as second-degree to sixth-degree pollution.

**Table 1:** Summary of the subjects enrolled in the PBMC expression profiling study

City	Gender		Age (year)		
	Female	Male	Median	Min	Max
BeiJing	15 (62.50%)	9 (37.50%)	29.5	23	48
TaiYuan	42 (80.77%)	10 (19.23%)	32	19	82
ShiJiaZhuang	35 (36.84%)	60 (63.16%)	43	21	80

We retrieved gene expression profiles of the peripheral blood mononuclear cells (PBMC) from the enrolled subjects. In order to control for the clonal heterogeneity of PBMC as well as other confounding factors in the transcriptome, we decomposed the expression matrix using a weighted clustering algorithm and yielded 18 co-expressed gene modules (Fig. 1c) [18]. Each module represents a unique biological process corresponding to specific cell types or exposure factors. Then we evaluated the association between each co-expressed gene module and the exposure levels of the five pollutants (Fig. 1d). As a result, the pollutants are separated into two different groups based on the response patterns of the gene modules. PM_2.5_, PM_10_ and SO_2_ cause very similar effects in gene expression, which differ from those of CO and NO_2_. In particular, we noticed two gene modules (“cyan” and “brown”) which are strongly associated with high exposure level of PM (PM_10_ and PM_2.5_, PCC > 0.7, Fig. 1c); and another two modules (“blue” and “turquoise”) strongly associated with low exposure level (PCC < − 0.7, Fig. 1c). As PM_2.5_ and PM_10_ cause similar effects in the transcriptome, we therefore refer both to “PM exposure” in the following analyses.

To reveal the biological background of the co-expressed modules strongly correlated with PM exposure, we annotated the modules for enriched KEGG pathways (Table 2 and Additional datasets: Data S1 and Fig. 1e). As a result, the module “cyan” significantly over represent the IL-17 signaling pathways (FDR = 0.000644); Cytokine-cytokine receptor interaction (FDR = 0.00114). The module “brown” instead, enriches for PD-L1 expression and PD-1 checkpoint pathway in cancer (FDR = 0.000397).

**Table 2:** Transcription factors (TF) underlying the co-expressed gene modules correlated with PM exposure (“cyan”, “brown”, “blue” and “turquoise”). TFs of which the binding motifs are significantly overrepresented in the co-expressed gene modules (FDR < 0.1); or the target genes are overrepresented in the eigen genes of each module (q-value < 0.05) are listed along with the KEGG pathways enriched (FDR < 0.05) in each module

Co-expressed Modules	Binding motifs enriched in upregulated genes	Transcription factor activities	KEGG Pathway enrichment
**cyan**	Nrf2(bZIP); NFkB-p65(RHD); n-Myc (bHLH); PIF4(bHLH); RAR:RXR (NR); IRF (bZIP,IRF)/Th17-BatF	NFKB1, RELA, JUN, NFIC, CEBPB, REL, CEBPD, SP1, CTNNB1, CDX1, STAT1, DLX1, STAT6, RELB, STAT3, DLX2, CEBPG, BCL6, PDX1, TFAP2A, STAT2, GATA4, JUNB, GATA1, JUND, CREB1, YY1, FOS, LEF1	Viral protein interaction with cytokine and cytokine receptor; IL-17 signaling pathway; Chemokine signaling pathway; Cytokine-cytokine receptor interaction; Salmonella infection; Rheumatoid arthritis; TNF signaling pathway; Fluid shear stress and atherosclerosis; Tuberculosis; NF-kappa B signaling pathway; AGE-RAGE signaling pathway in diabetic complications
**brown**	HOXA2(Homeobox); Hoxb4(Homeobox); STAT4(Stat);Pax7(Paired,Homeobox); E2A(bHLH); Stat3(Stat); STAT6(Stat)	MYC, SP1, TP53, STAT1, CEBPA, STAT3, HOXD3	PD-L1 expression and PD-1 checkpoint pathway in cancer
**blue**	FOXP1(Forkhead); Fli1(ETS); GABPA (ETS); GATA3(Zf);RUNX-AML (Runt); E2A(bHLH); ox2(HMG); ETS1(ETS); Eomes(T-box)	MYC, TFAP2A, SP1, ETS1, SPI1, GLI2, USF1, SP2, RELA, SP3, ELK1, POU2F2, REL, STAT3, POU1F1, ETV4, RELB, FOXO3, NFKB1, USF2, RUNX1, FOXO1, E2F1, CTNNB1, MYB, E2F6, MYBL2, RARA, GLI1, PPARD, JUN, RARG, PAX6, NFYA, TP53	Viral protein interaction with cytokine and cytokine receptor; ErbB signaling pathway; Epithelial cell signaling in Helicobacter pylori infection
**turquoise**	Fli1(ETS), ETS1(ETS); ELF1(ETS); Etv2(ETS); EABPA (ETS); Ets1-distal (ETS); RUNX (ETS,Runt)	CREB1, RBPJ, SP1, MYC, ATF1, CREBBP, SPI1, NFKB1, RELA, SREBF2, SREBF1, TP53, ETV4, E2F1, EGR1, FOXO1, NOTCH1, TFAP2A, YY1, BRCA1, BCL3, ETS1, TCF7L2, USF2, ATF6, JUN, STAT3, ESR1, CREM, PPARA, SMAD1, E2F6, FOXO3, PPARG, WT1, NFATC2, FLI1, RFX1, EGR4, OLIG1, FOXP1, CEBPB, PPARD, CTNNB1, ELK1, STAT5B, IRF9, STAT5A, ATF2, NFYA, GLI2, AR, USF1, STAT1, RELB, ID1, MYBL1, POU2F2, SMAD3, SMAD2, E2F4, ERG, FOXH1, CEBPA, REL, NFIC, ARNT, FEV, POU1F1, SP3, HOXA5, CEBPE, RARB, FOXO4, MYB, NFIA, NFATC1, SOX10, RARA, NR2F2, MITF, GABPA, HBP1, RARG, STAT2, SMAD4, HIF1A, NR1H2, NR1H3, TCF7, RUNX2, ATF4, NFE2L2, TBP, HNF4A, JUNB, NR2F1, CEBPD, GLI1, GATA1	Lysine degradation; RNA degradation; N-Glycan biosynthesis; Ubiquitin mediated proteolysis; Valine, leucine and isoleucine degradation; Spliceosome; RNA transport; Hepatitis C; Propanoate metabolism; Cytosolic DNA-sensing pathway

### Transcription factors mediate the inflammatory responses to PM exposure

Next, we seek to identify the transcription factors (TFs) that regulate the co-expression of genes in response to PM exposure. As the change in the expression levels of the TF is difficult to detect, we first assessed the transcription factor activities based on the enrichment of the corresponding target genes present in each co-expression modules (Table 2, and Additional datasets: Data S3) [19]. From the modules positively correlated with PM exposure, we retrieved we retrieved 29 (“cyan”) and 7 (“brown”) TFs of which the target mRNAs are significantly enriched (FDR < 0.1), including NFKB1 (“cyan”, FDR =0.00128), MYC (“brown”, FDR = 0.00147), STAT3 (“cyan”, FDR = 0.0192) etc. From the modules negatively correlated with PM exposure, we retrieved 35 (“blue”) and 100 (“turquoise”) significant TFs, including MYC (“blue”, FDR =0.0008621), CREB1 (“turquoise”, FDR = 0.0004673), SP1 (“turquoise”, FDR = 0.001402), ETS1 (“blue”, FDR = 0.003448; “turquoise”, FDR = 0.01028), RUNX (“blue”, FDR = 0.081) and so on.

To further reveal the transcription factors underlying the co-expressed modules, we resort to the DNA-binding-motifs which are bounded by known TFs and previously annotated in specific cell types [23]. For each module, we divide the member genes into two subsets, the “upregulated set” and the “down-regulated set”. We evaluated the enrichment of 388 TF-binding motifs in the DNase I hypersensitivity sites (DHS) in the poised cis-regulatory regions (Additional datasets: Data S2) of either set of genes (Additional datasets: Data S4). As a result, we identified several enriched DNA motifs from the co-expressed genes significantly correlated with PM exposure, which are bounded by specific transcription factors (q-value < 0.1, Tale 2). After removal of cell types that are not related to PBMC, we noticed several transcription factors that modulate the differentiation of T-cells. For example, the upregulated set of the module “cyan” overrepresents NRF2 motif (q-value = 0.0174) and IRF4/BATF co-localization motif (q-value = 0.0534); the upregulated set of the module “brown” overrepresents STAT3 (q-value = 0.0901), STAT4 (q-value = 0.0408) and STAT6 (q-value = 0.0903); the upregulated set of the module “blue” overrepresents FLI1 (q-value = 0.0215) and GATA3 (q-value = 0.0673). On the other hand, we find no known binding motifs enriched in the “down-regulated sets” of any co-expressed gene modules.

We compared the results of TF enrichment to the enrichment of pathways in the modules. As a result, we noticed six relevant transcription factors, which are likely regulating the co-expression in response to PM exposure. NFKB1 is enriched in module “cyan” for binding motif, target transcripts as well as the corresponding pathway. In the same module, we noticed another TF, IRF4 (with its binding partner BATF), of which the binding motif and the corresponding pathway (IL-17 signaling) are both significantly enriched. In module “brown”, we found that the binding motif and the target transcript of STAT3 are also significantly enriched. Finally, transcription factors FLI1, ETS1 and RUNX are enriched in module “blue” and module “turquoise” for both binding motif and the target genes. Among the six transcription factors, FLI1, ETS1 and RUNX are overrepresent in both modules that are negatively associated with PM exposure, hence the function is suppressed and are less likely to be the effector. On the other hand, although NFKB1 enrichment is associated with high PM exposure, it is known as a general regulator of inflammation and widely reported for its role in diverse immune responses [24–26]. Therefore, we figure that IRF4 and STAT3 are the likely effectors of PM exposure and focus on the two genes in the following function validations. STAT3 is reported as effectors of PM exposure by previous studies [12, 27–29], and both TFs are known regulators of T-cells [30–38].

### Exposure to PM influences T-cell polarization in vitro

The transcription factors enriched in the correlated co-expressed gene modules strongly suggests that PM exposure can influence the differentiation of T-cells. We then set out to further verify the effects of PM on T-cell differentiation.

Before the functional analysis, we analyzed the physical and chemical characteristics of the PM samples used for functional analysis. As for the chemical compositions, the major organic compositions of the particulate matters are organic carbon (OC) and SO_4_^2−^. The major metals are Ca, Fe, Al, Mg, K (Table S2). The average proportion of PM_2.5_ and PM_10_ in the PM sample is 0.75 (95%CI: 0.70–0.80) and 0.22 (95%CI: 0.17–0.28), respectively (Fig. S2A, B). Although prior study show that protease activity contributes to the immunogenicity of PM by induction of Th2 responses [39], there is no significant protease activity (1.08-fold of the blank control) in the PM samples we used at a concentration of 10 mg/ml (Fig. S2C).

**Fig. 2:**
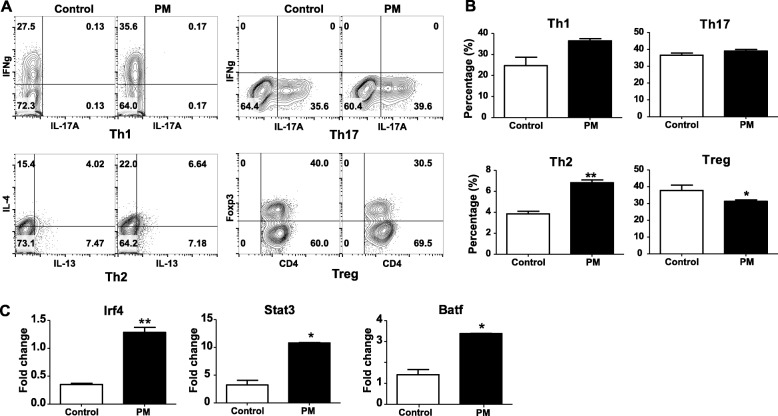
PM exposure alters T-cell polarization in vitro. **a**, flow cytometry analysis based on selected markers showing altered differentiation of Th1 (INFg, upper left), Th2 (IL4, IL13, lower left), Th17 (IL17, upper right) and Treg (FOXP3, lower right) in response to treatment with PM suspension (“PM”) as compared to control; **b**, the percentage of four T-cell subtypes with standard error are compared between PM-treated naïve T-cells and control; **c**, the expression levels of three predicted T-cell related transcription factors are compared between PM-treated naïve T-cells and control using qPCR (* *P* < 0.05, ** *P* < 0.01, *** *P* < 0.001)

We treated mouse-derived naive CD4+ T-cells with PM suspension and observed significantly increased polarization of T-helper cell type 2 (Th2, 77.04%, *P* = 0.00690), type 1 (Th1, 13.52%, *P* = 0.0580), and type 17 (Th17, 6.02%, *P* = 0.0926). On the other hand, the polarization of regulatory T-cells (T-reg) in the naive CD4+ T-cells decreases with PM treatment (16.67%, *P* = 0.0130) (Fig. 2).

We further assessed the expression levels of the transcription factors of which the binding motifs are overrepresented in the co-expressed gene modules positively correlated with PM exposure. As a result, the expression levels of Stat3, Irf4 and Batf significantly increase with PM treatment (*P* < 0.05, Fig. 2c).

The change in the expression levels of Stat3 and Irf4/Batf are consistent to the altered T-cell polarization in naïve CD4+ T-cells. Together we hypothesize that Stat3 and Irf4/Batf are the potential effectors of PM exposure and modulators of consequential T-cell polarization.

### Exposure to PM influences T-cell polarization in vivo

**Fig. 3:**
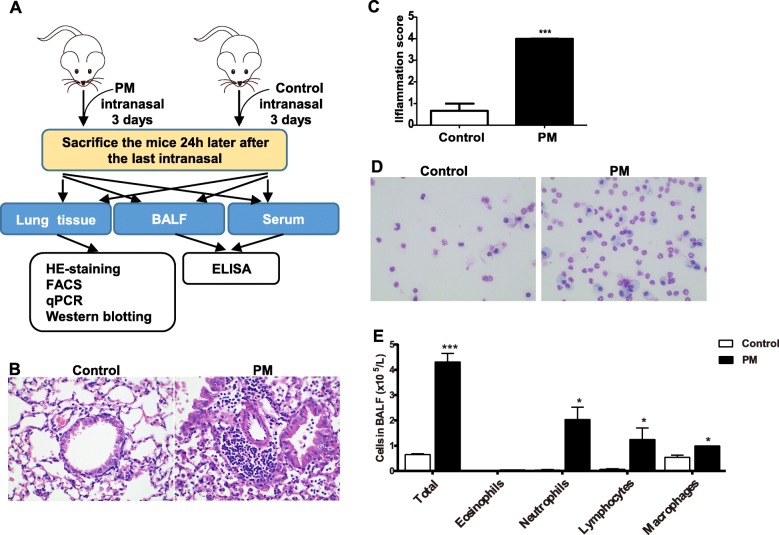
Intranausal PM exposure causes inflammatory responses in mouse lung in vivo. **a**, the schematic view of the animal experiment, each group of six BALB/c mice were treated with PM suspension for 3 days before the lung tissue, BALF and serum were collected and analyzed; **b**, inflammatory alterations are observed in mouse lung following intranosal treatment of PM suspension; **c**, comparison of the pathological inflammation score in mouse lung tissue with and without PM treatment; **d**, Giemsa stain of cells in BALF from mice with and without PM treatment; E, the number of inflammation cells in BALF, include eosinophils, neutrophils, lymphocytes and macrophages. (* *P* < 0.05, ** *P* < 0.01, *** *P* < 0.001)

To ascertain the impacts of PM exposure on T-cell polarization in vivo, we treated healthy mice (BALB/c) with PM suspension intranasally (Fig. 3a). In mouse lung treated with PM, we observed infiltration of neutrophils and lymphocytes into the terminal bronchiole and small vessels (Fig. 3b), which correspond to significantly increased inflammation score (*P* = 0.0006, Fig. 3c). Such pathological changes are similar to the inflammatory responses in allergic respiratory diseases such as asthma. In the bronchoalveolar lavage fluid, we observed consistent increase in the number of neutrophils, lymphocytes and macrophages (*P* < 0.05) but not eosinophils (Fig. 3d, e).

**Fig. 4:**
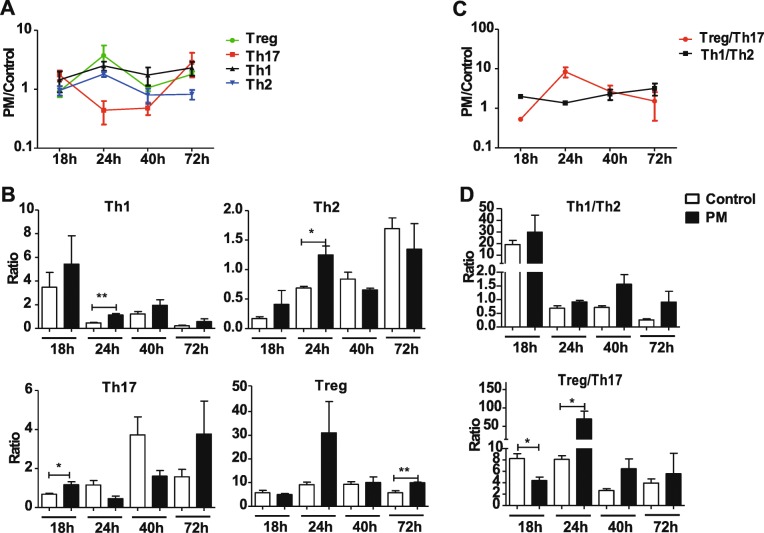
PM exposure alter T-cell polarization and cause Treg/Th17 imbalance in vivo. **a**, baseline-adjusted Th1, Th2, Th17 and Treg levels in mouse lung at 18 h, 24 h, 40 h and 72 h following PM treatment, respectively. Each treatment group contains six BALB/c mice; **b**, the Levels of each T-cell subtype are compared at each time point between PM-treated group and the control group; **c**, baseline-adjusted Th1/ Th2, Treg/Th17 ratios in mouse lung at 18 h, 24 h, 40 h and 72 h following PM treatment; **d**, the ratios of Th1/Th2, Treg/Th17 are compared between PM-treated group and the control group at each time point (* *P* < 0.05, ** *P* < 0.01, *** *P* < 0.001)

Then we assessed the polarization of Th1, Th2, Th17 and Treg in mouse lung at different time points following the treatment of PM suspension (Fig. 4a, see Additional file 1: Figure S3). At 18 h, we observed significantly elevated Th17 activities (70.87%, *P* = 0.0367); followed by increased activities of Th1 and Th2 (143.57%, *P* = 0.0069 for Th1 and 81.55%, *P* = 0.0228 for Th2) at the 24 h. Finally, at 72 h we noticed a significant Treg response and a restoration of Th17. We also assessed the balance between Th1/Th2 and Treg/Th17, respectively (Fig. 4b). As a result, Th1/Th2 balance remains relatively stable throughout 72 h. Treg/Th17 ratio drops to 0.532 at 18 h (*P* = 0.0221) then mount to 8.651 at the 24 h (*P* = 0.0442). The fluctuation in Treg/Th17 balance is mainly driven by the fast changing Th17 activities in the first 24 h following the treatment, which also causes of the pathological changes in the airway.

**Fig. 5:**
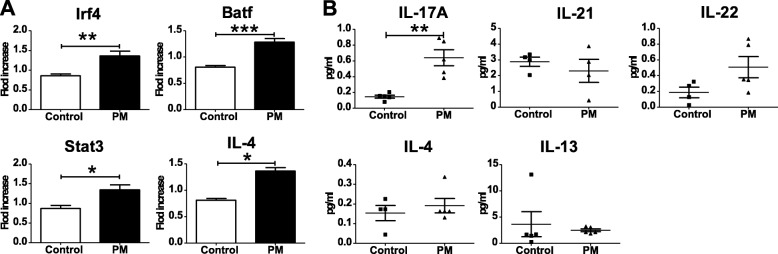
Predicted transcription factors of T-cell differentiation which are associated with PM exposure and the related cytokines change consistently in response to PM treatment in vivo. **a**, the expression levels of T-cell related transcription factors (Irf4, Batf4, Stat3) and IL-4 in mouse lung are measured using qPCR and compared between PM-treated group and the control group. Each treatment group contains six BALB/c mice; **b**, T-cell differentiation related cytokines in bronchoalveolar lavage fluid are measured using ELISA at 24 h following the treatment and compared between treatment group and control group (* *P* < 0.05, ** *P* < 0.01, *** *P* < 0.001)

Consistent to the changes in T-cell polarization, the expression levels of Stat3 (*P* = 0.0286), Irf4 (*P* = 0.0038) and Batf (*P* = 0.0002) are also significantly higher in the mouse lung following the treatment of PM suspension (Fig. 5a). We further assessed the expression levels of the related interleukins in the bronchoalveolar lavage fluid (BALF, Fig. 5b). As a result, the expression levels of IL17A (*P* = 0.0079) is elevated in following the treatment with PM suspension. The other two interleukins related to Th17, IL21 and IL22 however show no significant change. As for Th2 related interleukins in PM-treated group, we observed a significant increase of IL4 (*P* = 0.0268) in lung tissue, but no significant change of IL4 and IL13 levels in the BALF (Fig. 5b). Finally, in the mouse serum, we did not observe significant changes in the cytokine levels that relate to the T-cell subtypes aforementioned (see Additional file 1: Figure S4).

### Stat3 and Irf4 signaling is involved in the Th2 and Th17 differentiation induced by PM

**Fig. 6:**
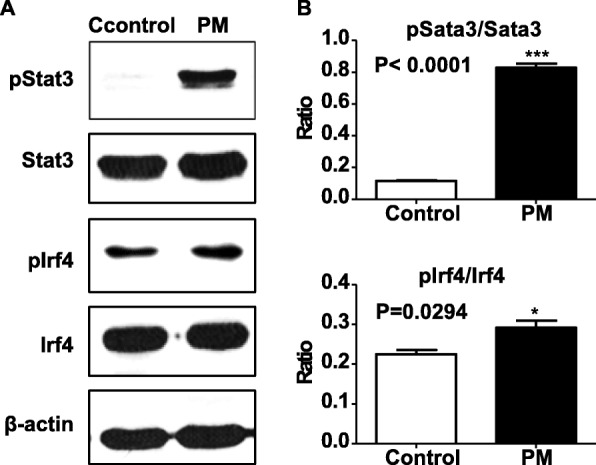
The levels of phosphorylated Stat3 and Irf4 increases in mice lung tissues in response to PM exposure in vivo. Two groups of six BALB/c mice were treated with PM suspension for 3 days then after 24 h the lung tissue were collected for Western Blot assay. **a**, Western Blot showing the protein expression levels of phosphorylate-Stat3 (pStat3), un-phospho-Stat3 (Stat3), phosphorylate-Irf4 (pIrf4), un-phospho-Irf4 (Irf4) with beta-actin as control; **b**, the ratios of phosphorylate-Stat3 versus un-phospho-Stat3 and phosphorylate-Irf4 versus un-phospho-Irf4 increase significantly in response to PM treatment (* *P* < 0.05, ** *P* < 0.01, *** *P* < 0.001)

Base on both in vitro and in vivo evidence, we figure that Stat3, Irf4 are potential effectors of PM exposure and directly involved in the altered T-cell polarization. Therefore, we moved on to verify that Stat3 and Irf4 functions in response to PM exposure at the protein level. Stat3 and Irf4 are both known transcription factors promoting Th2/Th17 polarization [40, 41]. We measured the protein levels of the activated form of Stat3 (phosphorylated-Stat3) and Irf4 (phosphorylated-Irf4) in the lung tissue following the treatment of PM suspension. As a result, phosphorylated Stat3 significantly increases in the lung tissues of mice 24 h following the treatment (612.03%, *P* < 0.001). And phosphorylated Irf4 levels also show significant elevation following the treatment of PM suspension (29.92%, *P* = 0.0294) (Fig. 6). These results, together, suggest that Irf4 and Stat3 are the main effectors of PM exposure thus modulate the immunogenicity of particulate matters in the respiratory tracts.

## Discussion

The hazardous effects of particulate matters on human health have been proved by prior studies from two aspects. First, populational studies evaluate the health effects from long-term survey confirm that exposure to PM is associated with diverse diseases. Then empirical studies use cell lines or animals to validate the acute effects of PM. Many of these studies suggest that the toxicity of PM is related to the immunogenicity [42], but few verified the biological mechanism in human subjects [43]. Here we conducted peripheral blood gene expression profiling in a healthy population in northern China. Our data shows that exposure to PM causes systematic effects in the transcriptome and we identified sets of co-expressed genes which strongly correlate with the level of exposure.

We then described a method integrating multi-level information to infer the transcription regulators driving the co-expressed gene modules strongly associated with PM. We report three transcription factors as effectors of PM exposure: NFkB and STAT3 are previously reported by in vitro analysis [44, 45]; IRF4, however, is reported for the first time to regulate in the immune responses to PM. IRF4 is known as a regulator of T-cell differentiation which is activated by NFkB, NFAT and TLR4 signaling [46–51]. Plus, IRF4 is involved in various inflammatory responses to microbial pathogens such as muramyl dipeptide (MDP) or lipopolysaccharide (LPS). Whether IRF4 is activated by PM through the same signaling modules still remain to be elucidated.

Our analyses demonstrate that the activation of Stat3 and Irf4 are associated with Treg/Th17 imbalance in response to PM treatment. The imbalance of Th17 and Treg is implicated in many allergic diseases in respiratory system [52, 53]. Especially, Th17 mediated inflammatory responses are crucial for the onset, exacerbation and control of Asthma and Chronic Obstructive Pulmonary Disease [54–59]. The role of STAT3 in Treg/Th17 imbalance in widely reported for allergic diseases. STAT3 activate Th17 upon IL-6 and IL-23R signaling and degrade by ubiquitination [60–62]. STAT3 is a known effector of PM [63–65]; IRF4 is also known for its function in Asthma by activating Th17 through regulation of chromatin accessibility but not for the role in response to PM exposure [66, 67]. Together our data suggest a potential risk of PM exposure to allergic respiratory diseases and regulators of Th17/Treg imbalance are likely effectors of PM.

There are other T-cell subtypes are also associated with the inflammatory responses to PM but lack of consistent evidences from in vitro validation in our data. For example, Treg activity decrease after 48 h in response to PM treatment in primary T cell culture but show no significant change in mice. Such inconsistent observations are attributed to the difference of in vitro and in vivo analysis, as the latter is influenced by more complexed effects and is more likely to represent the real biological activity. In addition, our data demonstrate the time-course of T-cell responses following the exposure to PM, which suggest the T cell responses reaches a steady state through an interactive process.

Quantification of the exposure level of PM in a real-world cohort is subject to various confounding effects from environmental to behavioral factors. In this study, we use three-week average concentration of the PM. As prior epidemiological studies observed the strongest effects of PM exposure between 5 to 21 days [68]. Moreover, many in vitro studies reveal the full effects of PM exposure on T-cell differentiation in a course of 2–3 weeks [69]. finally, a three-week lag also minimizes possible carry-over effects from previous exposure [70].

The expression profiles from PBMC consist transcripts from multiple cell types. Here we performed conformity-based decomposition of the expression profile to account for the latent heterogeneity and retrieved gene modules representing specific biological processes. While our studies predicted and validated some of the major effectors of PM exposure, there may be other effectors yet to be discovered from larger cohort.

The current study does not address the variations of the physical-chemical properties in the PMs from different sources, which eventually influence the immunogenicity of PM. The PM samples used in this study are mostly originated from combustion of fossil and biomass fuels. Some molecules such as the heavy metals, polycyclic aromatic hydrocarbons are absorbed by the particles and cause immune responses [71]. Protease activity is known to induce Th2 responses but doesn’t present in the samples we used. This can be caused by the sample collection and preparation. On the other hand, it also suggests that other factors contribute to the immunogenicity of the PM.

Finally, the roles of the T-cell regulators in response to PM need to be validated empirically in animal models with deficient STAT3 and IRF4. The current study highlights STAT3 and IRF4 as possible effectors of PM exposure and inform future functional analyses to reveal the biological and pathological background of PM caused respiratory allergy.

## Conclusions

In summary, we demonstrate the transcriptional effects of PM exposure in a healthy population and verified the corresponding regulatory activities of IRF4 and STAT3 using cell and animal models, which further inform the molecular basis of the immunogenicity and pathogenicity of PM. In addition, our results provide useful clues to clinical management for PM-associated allergic respiratory diseases.

## Supplementary information

**Additional file 1: **Reagents and Antibodies. **Table S1.** Primer sequences for qRT-PCR analyses. We designed all the primers using *https://sg.idtdna.com/pages/products/custom-dna-rna* combine with the *NCBI* and *UCSC* websites**. Table S2.** Chemical characteristics of the PM samples used for functional analyses. Water soluble ions were analyzed by ion chromatography, element measurement by inductivity coupled plasma-mass spectrometry (ICP-MS); and organic carbon (OC) and elemental carbon (EC) fractions by DRI model 2001 carbon analyzer. **Figure S1.** The average concentration of PM_2.5_ and PM_10_ in the three Chinese cities (Beijing, Taiyuan, Shijiazhuang) during the study period. We collected environmental data of 3 weeks preceding the sampling. The actual time period of sampling in the three cities: 10/04/2014–26/04/2014 in Shijiazhuang, Taiyuan: 21/02/2014–01/05/2014 and Beijing: 26/09/2014–16/10/2014. **Figure S2.** Size distribution and protease activity of the PM sample. A. 20 × microscopic view of PM suspension showing the distribution of PM_2.5_ and PM_10_; B. protease activity of the PM samples based on FITC-labeled casein cleavage hydrolysis assay. The protease activities are measured by mean fluorescence level with standard deviation. **Figure S3.** Expression levels of T-cell related cytokines in mouse serum follow the treatment of PM. **Figure S4.** Differentiation statuses of T-cell subtypes in mouse lung following the treatment of PM suspension. T-cell differentiation is evaluated by flow cytometry at 18 h, 24 h, 40 h and 72 h after the treatment.

**Additional file 2: Sheet data S1.** Gene list of the 18 co-expressed gene modules identified from the PBMC profiling study. **Sheet data S2.** KEGG pathways and GO biological processes significantly enriched in the co-expressed gene modules. **Sheet data S3.** Enrichment of the target genes of known transcription factors in the four modules strongly correlated with PM exposure. **Sheet data S4.** Enrichment of the binding motifs of known transcription factors in the poised cis-regulatory regions of the genes of the four modules strongly correlated with PM exposure.

## Data Availability

The datasets generated and analysed during the current study are available from the correspondence author on reasonable request.

## Abbreviations

PM: Particulate matter
COPD: Chronic obstructive pulmonary disease
AD: Atopic dermatitis
TF: Transcription factors
UFP: Ultra-fine particles
DHS: DNaseI hypersensitivity sites
TSS: Transcription starting sites
CMC: Carboxymethylcellulose
XMULAC: Xiamen University Laboratory Animal Center
WGCNA: Weighted gene co-expression network analysis
BALF: Bronchoalveolar lavage fluid
MDP: Muramyl dipeptide
LPS: Lipopolysaccharide
PBMCs: Peripheral blood mononuclear cells
SPF: Specific pathogen-free
