# Supplementary material for: IRF4 and STAT3 activities are associated with the imbalanced differentiation of T-cells in responses to inhalable particulate matters
Source: Respir Res. 2020 May 24;21:123. doi: 10.1186/s12931-020-01368-2 (PMC7245756; doi:10.1186/s12931-020-01368-2)
Supplement: Supplementary file 1 — Additional file 1: Reagents and Antibodies. Table S1. Primer sequences for qRT-PCR analyses. We designed all the primers using https://sg.idtdna.com/pages/products/custom-dna-rna combine with the NCBI and UCSC websites. Table S2. Chemical characteristics of the PM samples used for functional analyses. Water soluble ions were analyzed by ion chromatography, element measurement by inductivity coupled plasma-mass spectrometry (ICP-MS); and organic carbon (OC) and elemental carbon (EC) fractions by DRI model 2001 carbon analyzer. Figure S1. The average concentration of PM2.5 and PM10 in the three Chinese cities (Beijing, Taiyuan, Shijiazhuang) during the study period. We collected environmental data of 3 weeks preceding the sampling. The actual time period of sampling in the three cities: 10/04/2014–26/04/2014 in Shijiazhuang, Taiyuan: 21/02/2014–01/05/2014 and Beijing: 26/09/2014–16/10/2014. Figure S2. Size distribution and protease activity of the PM sample. A. 20 × microscopic view of PM suspension showing the distribution of PM2.5 and PM10; B. protease activity of the PM samples based on FITC-labeled casein cleavage hydrolysis assay. The protease activities are measured by mean fluorescence level with standard deviation. Figure S3. Expression levels of T-cell related cytokines in mouse serum follow the treatment of PM. Figure S4. Differentiation statuses of T-cell subtypes in mouse lung following the treatment of PM suspension. T-cell differentiation is evaluated by flow cytometry at 18 h, 24 h, 40 h and 72 h after the treatment. [file 12931_2020_1368_MOESM1_ESM.zip › revision_Additional file 1.docx]

**Supporting Information**

**IRF4 and STAT3 Play a Role in the Imbalanced Differentiation of T-Cells in Responses to Particulate Matters**

Jinzhun Wu, Dandan Ge, Taoling Zhong, Zuojia Chen, Ying Zhou, Lingyun Hou, Xiaoliang Lin, Jiaxu Hong, Kuai Liu, Hui Qi, Chaoying Wang, Yulin Zhou, Cheng Li, Chuan Wu, Shuiping Wu, Zuguo Liu, Qiyuan Li

**Reagents and Antibodies.**

The EastepTM Total RNA Extraction Kit and GoTaq® qPCR Master Mix kit were obtained from Promega. The Ovalbumin OVA, Aluminium hydroxide (AL(OH)_3_) and TEMED were purchased from Sigma-Aldrich. The Strong RIPA lysate and 30% Acr-Bis were purchased from Boster. The protein phosphatase inhibitor complex and Protease inhibitors were purchased from Roche. The BCA protein quantitative kit, CDNA synthesis kit and TRIzol Total RNA Extraction Kit was purchased from Tiangen. The film was purchased from Keda. The ECL chemiluminescence detection kit were purchased from Advansta. The Color prestain of molecular proteins marker was purchased from Thermo Fisher Scientific. The PVDF membrane was purchased from EMD Millipore. The 4 × loading buffer (contain DTT) was purchased from Solarbio. Cytokine levels were made using the ‘Meso Scale Discovery’ (MSD)-based multi cytokine approach.

The IRF4 (D9P5H) Rabbit mAb, phospho-Stat3 (Tyr705) (M9C6) Mouse mAb and Stat3 (124H6) Mouse mAb were purchased from Cell Signaling Technology. The phospho-IRF4 (Phospho-Tyr122/125) antibody was purchased from SAB Signalway Antibody. The Mouse β-Actin antibody was purchased from R&D. The Goat Anti-Rabbit IgG and Goat Anti-Mouse IgG were purchased from Boster. Oligo nucleotides were synthesized by Sangon Biotech (See [Supplemental Material Table S1](https://www.ncbi.nlm.nih.gov/pmc/articles/PMC4618176/#SD1) qRT-PCR primers).

**Flow Cytometry of Cell Culture**

Single naïve CD4+ T cells isolated from the spleen of mice aged 6-8 weeks were purified by CD4 (L3T4) MicroBeads and their purity was verified (>90%) by flow cytometry. Isolated CD4+ T cells were cultured in RPMI containing 10% FBS, 100 IU/ml penicillin, 100μg/ml streptomycin, 1mM sodium pyruvate, non-essential amino acids, 50μM β-mercaptoethanol, and 2 mM L-glutamine (R-10). Isolated CD4^+^ T-cells were stimulated with 5μg of plate-bound anti-CD3 and 1μg/ml of soluble anti-CD28 for 3 days in 48-well plates. Then we added 100μg PM in each well and corresponding stimulating factors to induce cell differentiation. Th1: IL-12 (20 ng/ml, R&D); Th2: IL-4 (20 ng/ml Miltenyi Biotec); Th17: IL-6 (20 ng/ml; Miltenyi Biotec); TGF-β (2 ng/ml; Miltenyi Biotec); Treg: TGF-b (5 ng/ml, Miltenyi Biotec). Cells were then incubated for 48 hours. Until 6 hours before harvest, PMA (20 ng/ml), ionomycin (500 ng/ml), and Golgi Stop (2 ul/well) were added.

The target cells were stained with the indicated fluorescence-conjugated antibodies for 20 minutes, washed then resuspended with 1% BSA/PBS flow cytometry assays (FACS) staining buffer containing DAPI (Invitrogen). Stained cells were analyzed with a BD LSRFortessa™ flow cytometer (BD Biosciences). Flow cytometry data were plotted and quantified using median fluorescence intensity (MFI) by FlowJo software (Treestar). The fluorescence-conjugated antibodies, anti-CD3ε (145-2C11), anti-CD62L (MEL14), anti-CD11b (M1/70), anti-B220 (RA3-6B2), anti-CD44 (IM7), anti-F4/80 (BM8) and anti-Gr-1 (RB6-8C5) antibodies, were from Biolegend (San Diego) or BD Biosciences.

**Blood, BALF and Lung tissue**

0.5ml-0.8ml eyeball blood was collected 24h after intranasal PM treatment and clotted at room temperature for 2-4 hours. Then serum is isolated by and stored at minus 80°C. After the collection of eyeball blood, tracheotomy was performed and a cannula was inserted into the trachea. Ice-cold SALINE (1ml) was instilled into the lungs, and BAL fluid was collected. Then centrifuged and aliquots of supernatant were stored at minus 80°C for ELISA. The cell precipitates in BALF were re-suspended with PBS, by counted and smeared. After Swiss-Giemsa staining following smeared, 300 cells per smear were counted for cell classification per smear [[1](#_ENREF_1)]. Lung tissue was exhausted bubbles with formaldehyde followed by immobilized, paraffinized.

**Lung Histopathology Staining**

Paraffin-embedded lung sections (5μm) were stained with hematoxylin and eosin (H&E). Cellular infiltration around the blood vessels and airways was scored to evaluate the lung inflammation according to the following criteria: 0, no infiltrates; 1, few inflammatory cells; 2, a ring of inflammatory cells 1 cell layer deep; 3, a ring of inflammatory cells 2 to 4 cells deep; and 4, a ring of inflammatory cells greater than 4 cells deep [[2](#_ENREF_2)]. A composite score was determined by adding the inflammatory scores for both vessels and airways.

**Flow Cytometry of Lung Tissue**

We sacrifice the mice at 18h, 24h, 40h, and 72h after the last nasal drip. Then we harvest the lung tissue and collect the lymphocyte through collagenase digesting, grind, and 70%/42% percoll separation. Then 1ug/ml Golgi Stop was used for blocking cytokine secretion. After 5 hours, we collected the cells for surface markers FACS staining at a ratio of 1:200 (anti-CD4, anti-CD8) for 15min. Fix Buffer/Permeabilization Buffer with 1:3 ratio was added at 4℃ for 30min. Finally, we stained Intracellular and intranuclear cytokine using antibodies at a ratio of 1:100 (anti-IFNg, anti-IL4, anti-IL-17A, anti-Foxp3) for 15min.

**ELISA**

To quantify cytokines expression *in vitro*, supernatants from differentiated T cells were collected on day 3 of culture and analyzed by capture ELISA. To quantify cytokines expression *in vivo*, we collected the eyeball blood and bronchoalveolar lavage fluid (BALF) from mice treated with PM suspension. Then, TGF-β, TNF-α, IL-6, IFN-g, IL-4, IL-17A, IL-13, IL-21, IL-33 and IL-22 levels were analyzed using mouse quantikine ELISA kits following the standard protocol (R&D).

**Quantitative Real-Time PCR**

Total RNA was isolated from lung tissue and treated with DNAse (Qiagen) following the manufacture’s instruction. cDNA synthesis was performed from 2µg of total RNA with . Quantitative real-time PCR was performed using SYBR green-based reagents on the ViiA 7 Real-Time PCR System (Life Technologies) with primer pairs targeting the cDNAs of *Rora, Rorc,* *β-actin,* et.al transcripts (see table S1). All qPCR reactions were run in duplicates and the resulted CT values were normalized to β-actin as a fold-induction over controls. All the primer sets were listed in supplemental material

**Western Blotting**

Ground lung tissue was lysed in RIPA buffer) and Complete Protease Inhibitors and Protein Phosphatase Inhibitor Complex (Roche). Then the protein concentrations in the lysates were determined by BCA assays. 20 to 80μg of proteins was separated on an SDS-10% polyacrylamide gel electrophoresis gel and transferred to polyvinylidene difluoride membranes by electroblotting. Immunoreactive proteins were visualized with ECL method (Pierce) and imaged by a Fujifilm LAS-4000 imager.

**Experimental statistical analysis**

Statistical analysis was performed with GraphPad Prism 5.0 and Excel version 14.4.7. One-way analysis of variance (ANOVA) with Tukey’s multiple comparison test was performed to compare test groups, as appropriate. While two tailed, unpaired Student’s t test was performed when only two test group were included. A p value of less than 0.05 was considered statistically significant. P values are indicated as follows: *P < 0.05; **P < 0.01; ***P < 0.001. Additional methods can be found in SI Material and Methods.

**Reference**

1. Guo HW, Deng JG, Yun CX, Hou GH, Jun DU: **Mechanism of Mangiferin Inhibiting the Airway Inflammation in a Murine Model of Asthma.** *Chinese Journal of Experimental Traditional Medical Formulae* 2012.

2. Ford JG, Rennick D, Donaldson DD, Venkayya R, Mcarthur C, Hansell E, Kurup VP, Warnock M, Grünig G: **IL-13 and IFN-γ: Interactions in Lung Inflammation.** *Journal of Immunology* 2001, **167:**1769-1777.

**Table S1.**

Primer sequences for qRT-PCR analyses. We designed all the primers using *https://sg.idtdna.com/pages/products/custom-dna-rna* combine with the *NCBI* and *UCSC* websites**.**

| **Primer** | **Forword** | **Reverse** |
| --- | --- | --- |
| Irf4 | GGTGTACAGGATTGTTCCAGAG | GAGCCATAAGGTGCTGTCAT |
| Batf | GGAGAGAGAAGAATCGCATCG | TTTCTCCAGGTCCTCACTCT |
| Stat3 | GTCTGTGACCAGACAGAAGATG | CGTACTCCATTGCTGACAAGA |
| Tnf | ACCACGCTCTTCTGTCTACT | AGGGTCTGGGCCATAGAA |
| IL4 | CAGAGACTCTTTCGGGCTTT | GCATGATGCTCTTTAGGCTTTC |
| Stat6 | GAAGGCTTCAGCATCGAGTAA | ACCATTGACAGGAGGGTCTA |
| Gata3 | GATGTAAGTCGAGGCCCAAG | TAGTGCCCGGTACCATCT |
| Tbet | CAACCAGCACCAGACAGAGAT | ACCAAGACCACATCCACAAAC |
| Foxp3 | CAGCTCTACTCTGCACCTTC | CACTTGCAGACTCCATTTGC |
| IL6 | TGGGAAATCGTGGAAATGAG | CTCTGAAGGACTCTGGCTTTG |
| Rora | GGAAGAGCTCCAGCAGATAAC | GGCAAACTCCACCACATACT |
| Rorc | GGGACAAGTCATCTGGGAT | GACGCGTGCAGGAGTAG |
| β-actin | GTGCTATGTTGCTCTAGACTTCG | ATGCCACAGGATTCCATACC |

**Table S2.**

Composition analysis results of PM, used in our experiment. Water soluble ions were analyzed by ion chromatography; element measurement was test by inductivity coupled plasma – mass spectrometry (ICP-MS); and organic carbon (OC) and elemental carbon (EC) fractions analyze by a DRI model 2001 carbon analyzer.

|  | **Mean（ug/m^3^)** | **SD** |
| --- | --- | --- |
| PM_10_ | 32.92 | 6.57 |
| Cl⁻ | 0.17 | 0.30 |
| NO₃⁻ | 0.85 | 0.38 |
| SO₄²⁻ | 1.73 | 0.61 |
| Na⁺ | 0.43 | 0.14 |
| NH₄⁺ | 0.36 | 0.29 |
| Mg²⁺ | 0.05 | 0.01 |
| Ca²⁺ | 0.68 | 0.17 |
| K⁺ | 0.11 | 0.04 |
| OC（organic carbon） | 4.32 | 1.46 |
| EC（elemental carbon） | 1.61 | 0.49 |
| **Element** | **Mean（ng/m^3^)** | **SD** |
| Be | 0.22 | 0.11 |
| Mg | 260.43 | 87.79 |
| Al | 379.00 | 182.54 |
| K | 186.45 | 87.60 |
| Ca | 736.54 | 397.51 |
| V | 14.85 | 6.23 |
| Cr | 7.39 | 7.12 |
| Mn | 12.99 | 7.11 |
| Fe | 497.38 | 280.46 |
| Ni | 8.00 | 2.95 |
| Cu | 5.92 | 2.08 |
| Zn | 64.54 | 41.69 |
| As | 0.79 | 0.52 |
| Se | 0.60 | 0.48 |
| Ag | 0.04 | 0.03 |
| Cd | 0.16 | 0.13 |
| Ba | 11.00 | 4.99 |
| Pb | 9.51 | 5.04 |


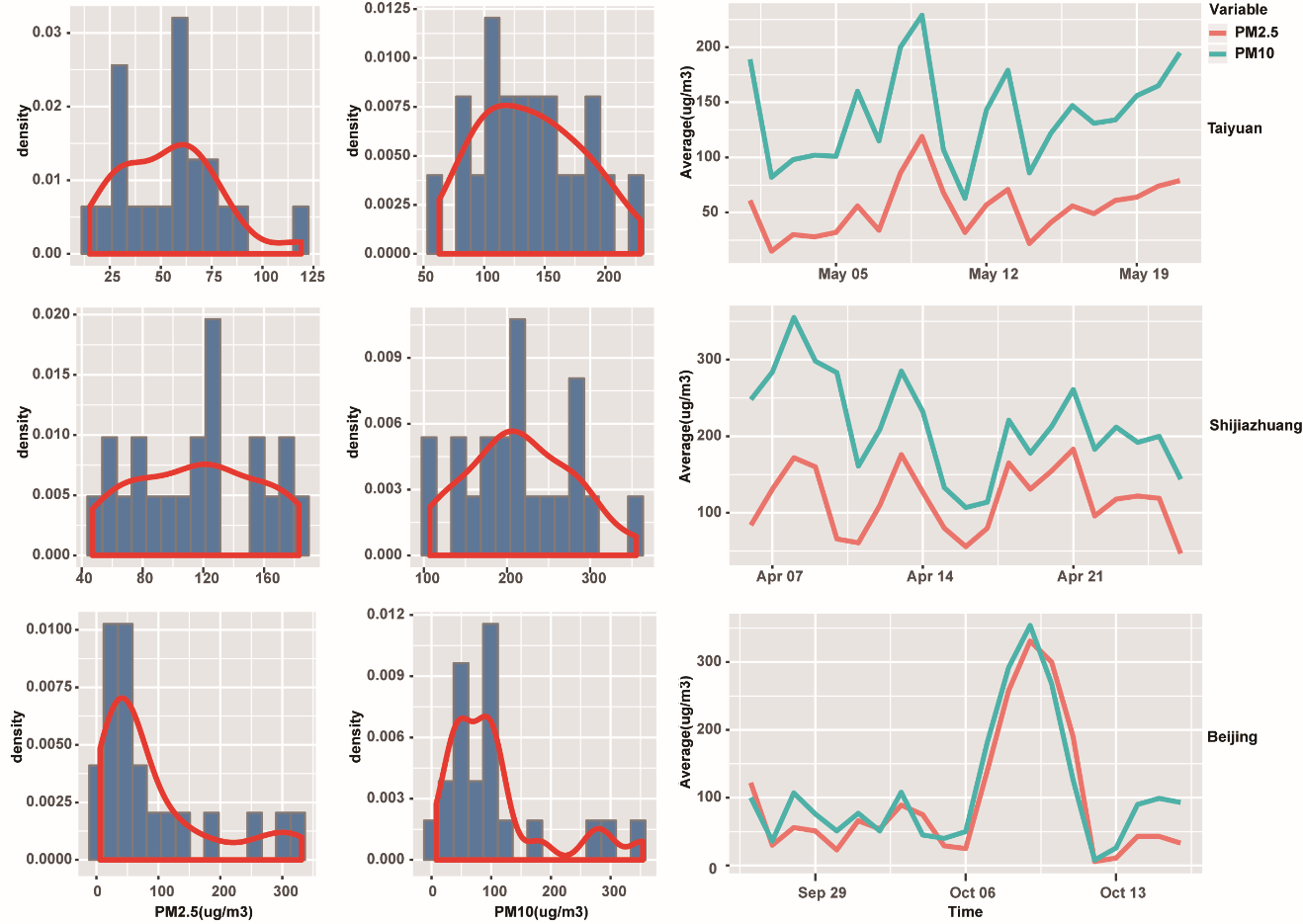


**Figure. S1.** The average concentration of PM_2.5_ and PM_10_ in the three cities during the study period. We collected environmental data of 3 weeks before the sampling. The actual time period of sampling in the three cities: 10/04/2014-26/04/2014 in Shijiazhuang, Taiyuan: 21/02/2014-01/05/2014 and Beijing: 26/09/2014-16/10/2014.


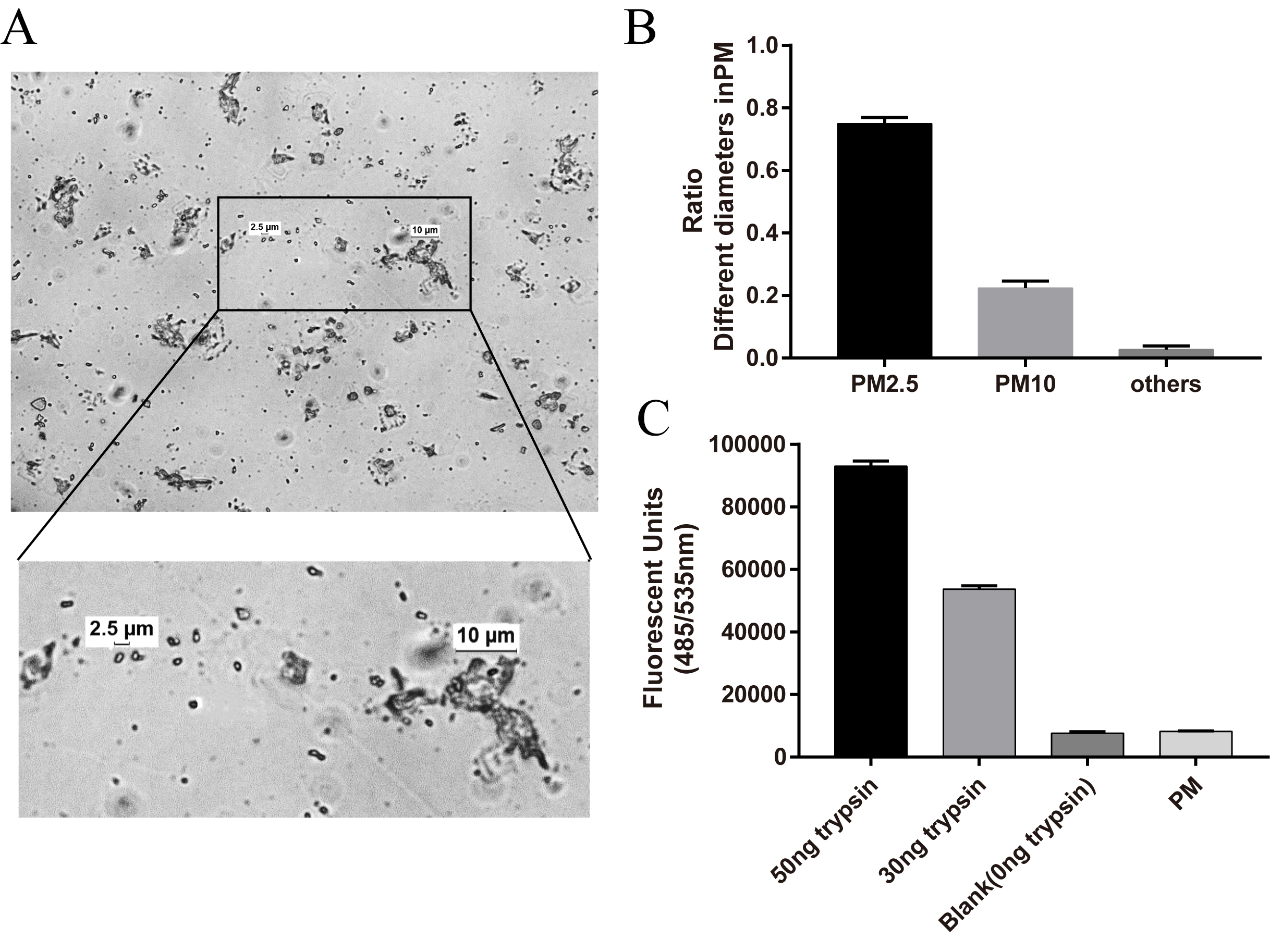


**Figure S2.** Size distribution and protease activity of the PM sample. A. 20x microscopic view of PM suspension showing the distribution of PM_2.5_ and PM_10_. B. protease activity of the PM samples based on FITC-labeled casein cleavage hydrolysis assay. The protease activities are measured by mean fluorescence level with standard deviation.


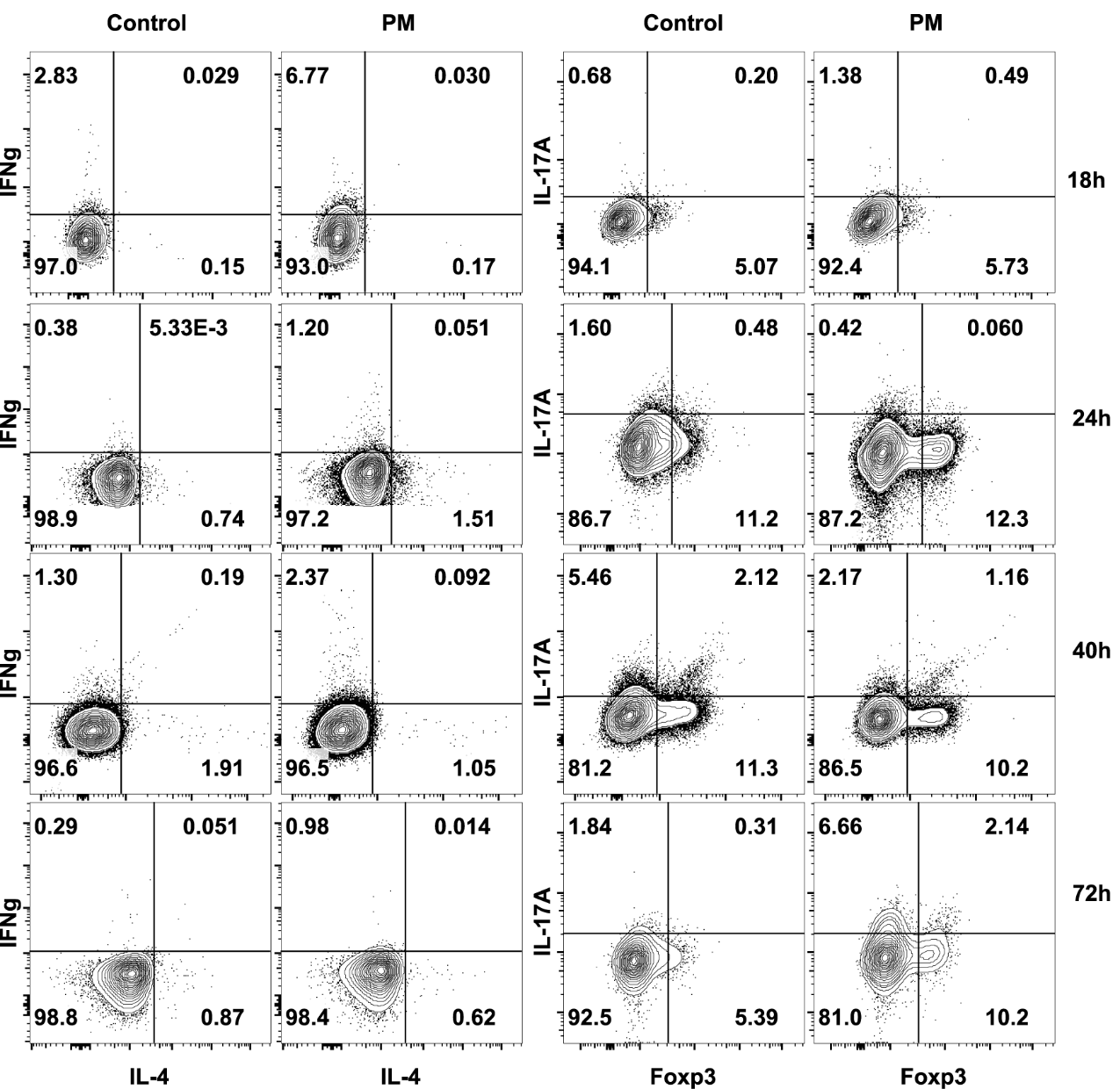


**Figure. S3.** Differentiation statuses of T-cell subtypes in mouse lung following the treatment of PM. T-cell differentiation is evaluated by flow cytometry 18h, 24h, 40h and 72h after the treatment.


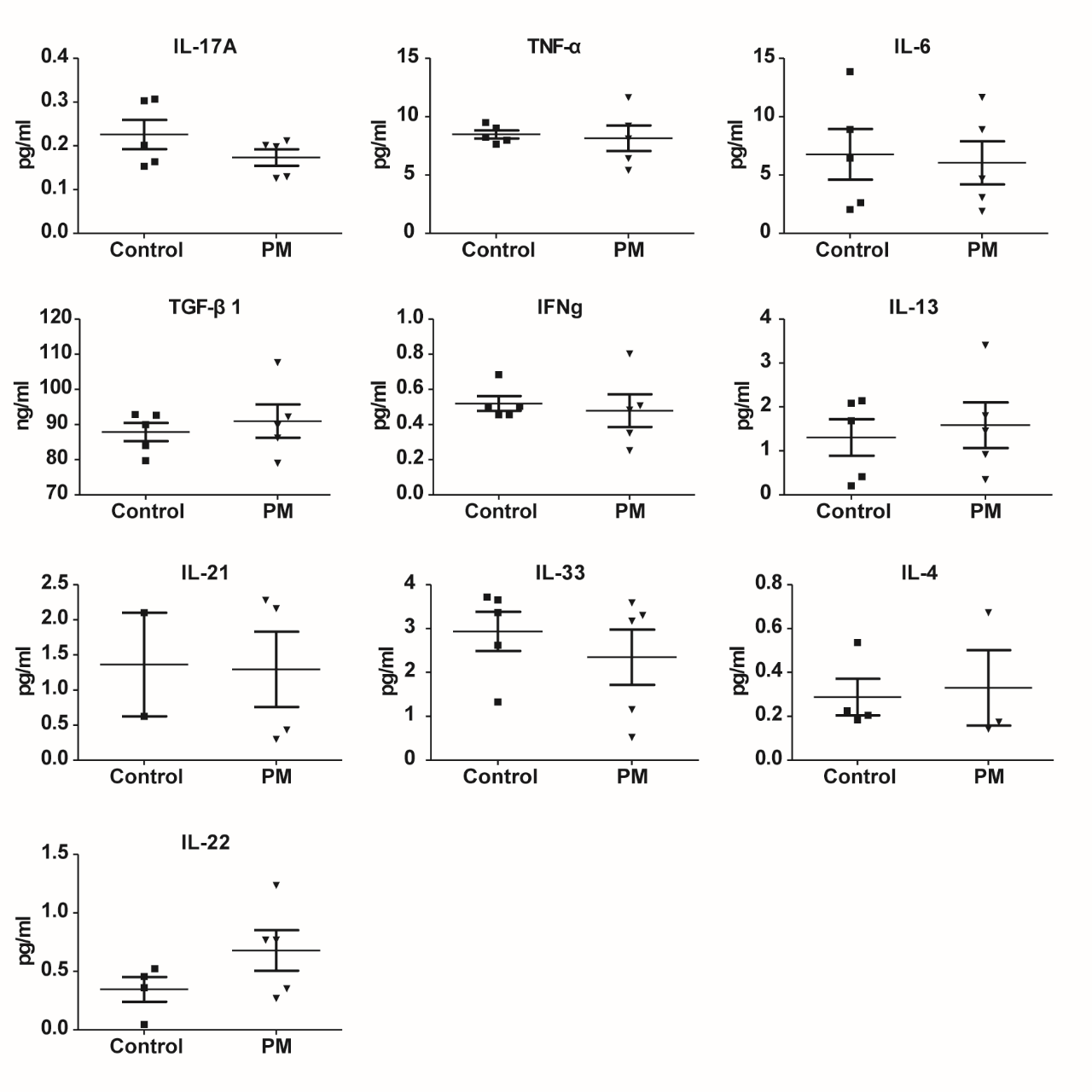


**Figure. S4.** Expression levels of T-cell related cytokines in mouse serum follow the treatment of PM.
