# Supplementary figures and images for: IRF4 and STAT3 activities are associated with the imbalanced differentiation of T-cells in responses to inhalable particulate matters
Source: Respir Res. 2020 May 24;21:123. doi: 10.1186/s12931-020-01368-2 (PMC7245756; doi:10.1186/s12931-020-01368-2)

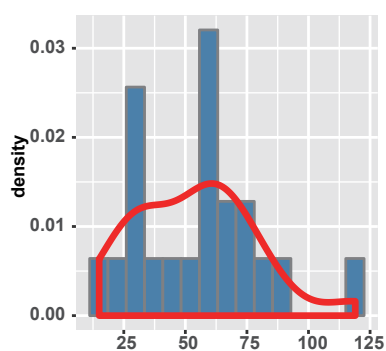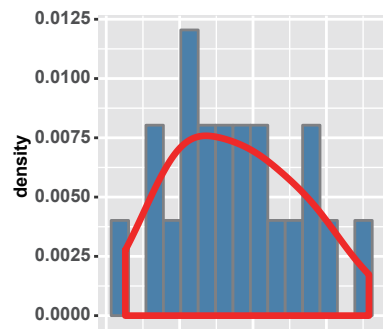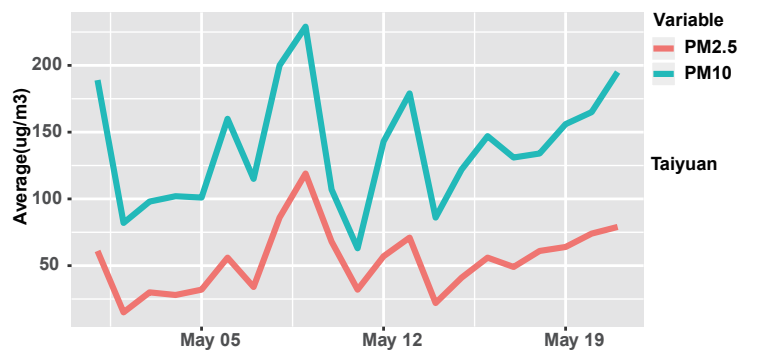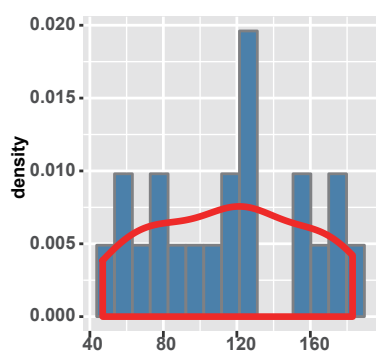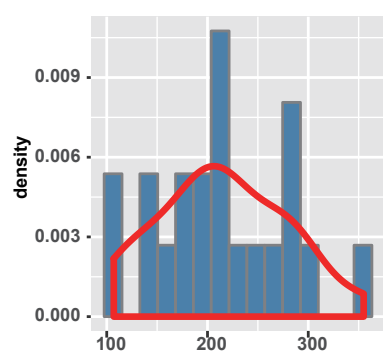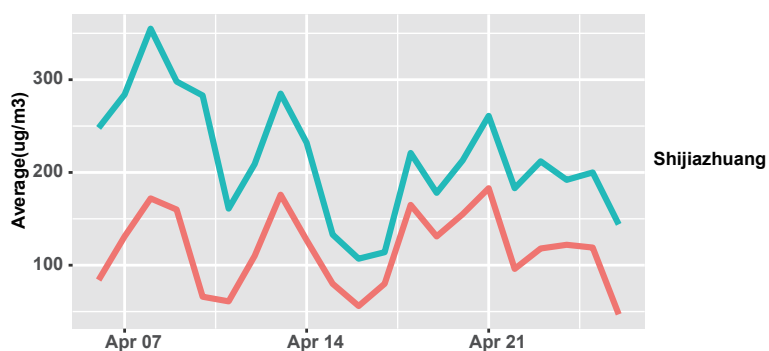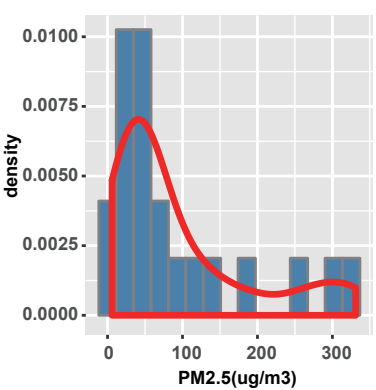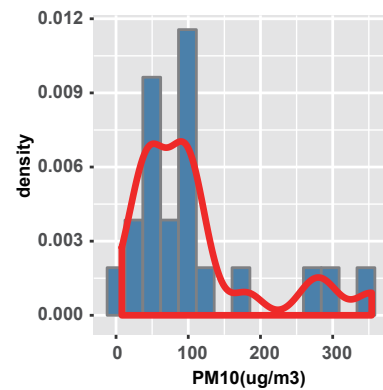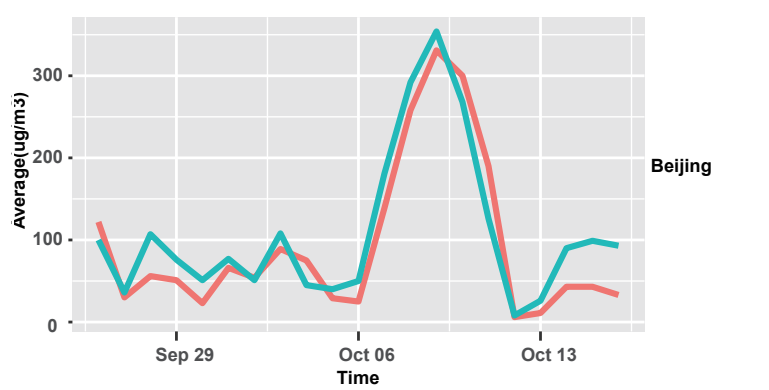

Supplement: Supplementary file 1 — Additional file 1: Reagents and Antibodies. Table S1. Primer sequences for qRT-PCR analyses. We designed all the primers using https://sg.idtdna.com/pages/products/custom-dna-rna combine with the NCBI and UCSC websites. Table S2. Chemical characteristics of the PM samples used for functional analyses. Water soluble ions were analyzed by ion chromatography, element measurement by inductivity coupled plasma-mass spectrometry (ICP-MS); and organic carbon (OC) and elemental carbon (EC) fractions by DRI model 2001 carbon analyzer. Figure S1. The average concentration of PM2.5 and PM10 in the three Chinese cities (Beijing, Taiyuan, Shijiazhuang) during the study period. We collected environmental data of 3 weeks preceding the sampling. The actual time period of sampling in the three cities: 10/04/2014–26/04/2014 in Shijiazhuang, Taiyuan: 21/02/2014–01/05/2014 and Beijing: 26/09/2014–16/10/2014. Figure S2. Size distribution and protease activity of the PM sample. A. 20 × microscopic view of PM suspension showing the distribution of PM2.5 and PM10; B. protease activity of the PM samples based on FITC-labeled casein cleavage hydrolysis assay. The protease activities are measured by mean fluorescence level with standard deviation. Figure S3. Expression levels of T-cell related cytokines in mouse serum follow the treatment of PM. Figure S4. Differentiation statuses of T-cell subtypes in mouse lung following the treatment of PM suspension. T-cell differentiation is evaluated by flow cytometry at 18 h, 24 h, 40 h and 72 h after the treatment. [file 12931_2020_1368_MOESM1_ESM.zip › figure_S1.pdf]

A

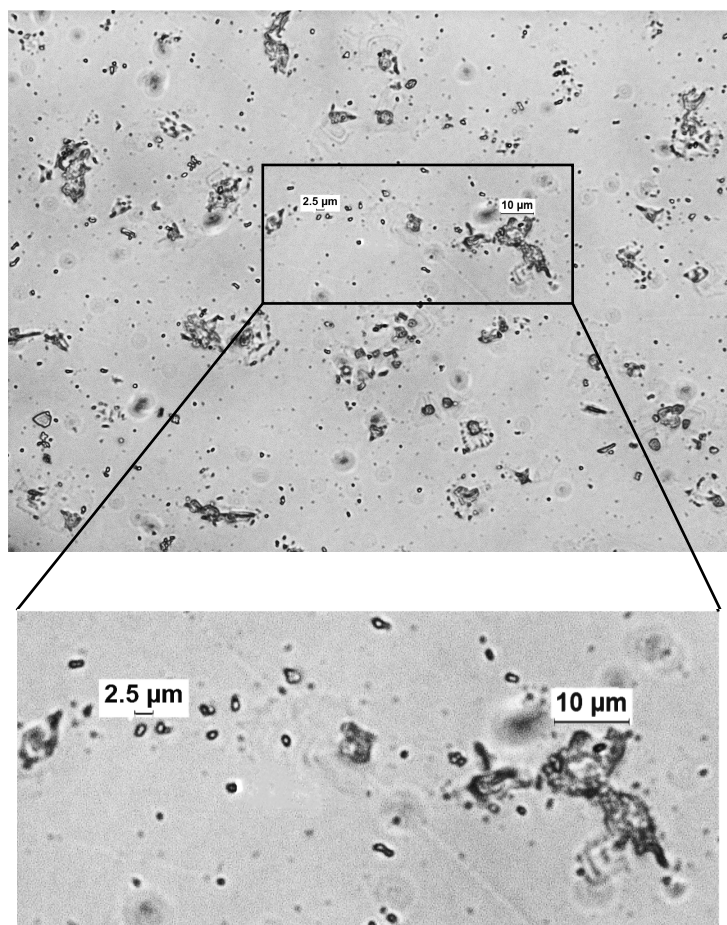

B

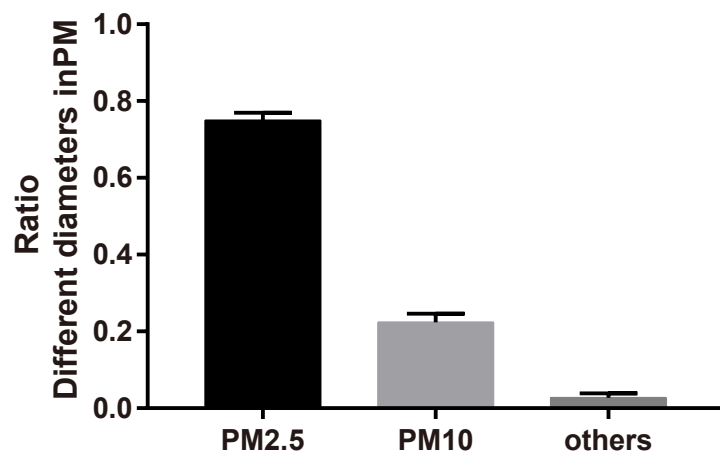

C

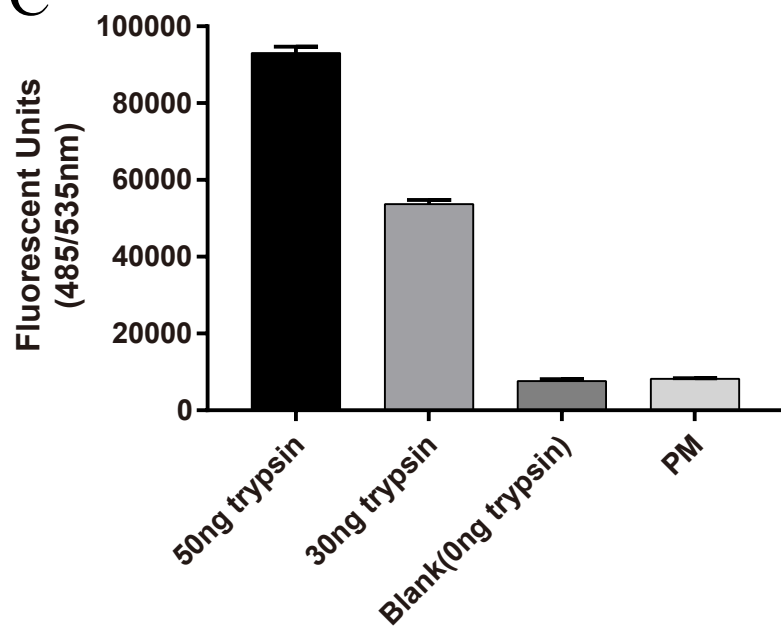

Supplement: Supplementary file 1 — Additional file 1: Reagents and Antibodies. Table S1. Primer sequences for qRT-PCR analyses. We designed all the primers using https://sg.idtdna.com/pages/products/custom-dna-rna combine with the NCBI and UCSC websites. Table S2. Chemical characteristics of the PM samples used for functional analyses. Water soluble ions were analyzed by ion chromatography, element measurement by inductivity coupled plasma-mass spectrometry (ICP-MS); and organic carbon (OC) and elemental carbon (EC) fractions by DRI model 2001 carbon analyzer. Figure S1. The average concentration of PM2.5 and PM10 in the three Chinese cities (Beijing, Taiyuan, Shijiazhuang) during the study period. We collected environmental data of 3 weeks preceding the sampling. The actual time period of sampling in the three cities: 10/04/2014–26/04/2014 in Shijiazhuang, Taiyuan: 21/02/2014–01/05/2014 and Beijing: 26/09/2014–16/10/2014. Figure S2. Size distribution and protease activity of the PM sample. A. 20 × microscopic view of PM suspension showing the distribution of PM2.5 and PM10; B. protease activity of the PM samples based on FITC-labeled casein cleavage hydrolysis assay. The protease activities are measured by mean fluorescence level with standard deviation. Figure S3. Expression levels of T-cell related cytokines in mouse serum follow the treatment of PM. Figure S4. Differentiation statuses of T-cell subtypes in mouse lung following the treatment of PM suspension. T-cell differentiation is evaluated by flow cytometry at 18 h, 24 h, 40 h and 72 h after the treatment. [file 12931_2020_1368_MOESM1_ESM.zip › revision_figure_S2.pdf]

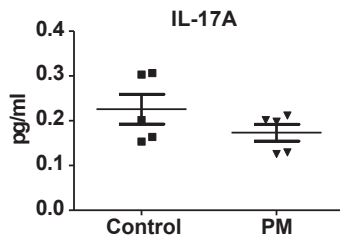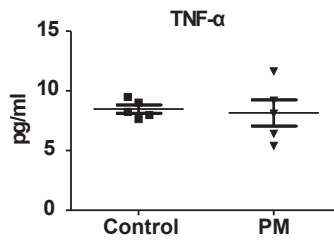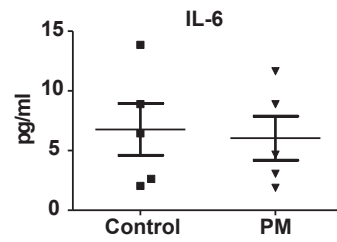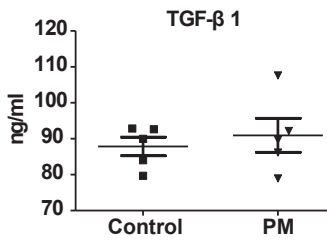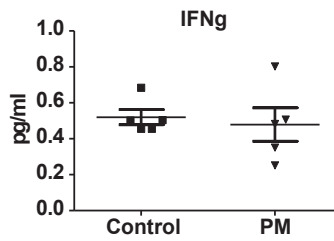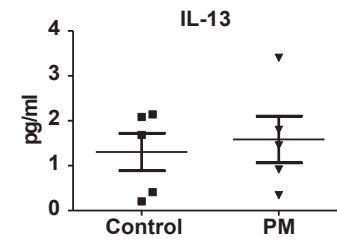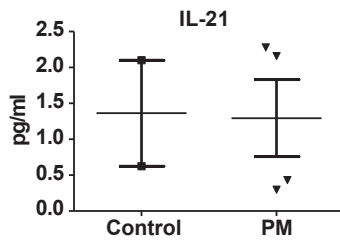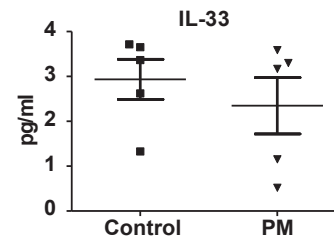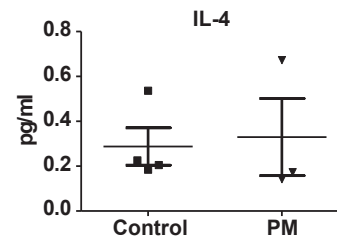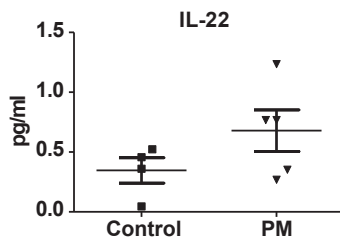

Supplement: Supplementary file 1 — Additional file 1: Reagents and Antibodies. Table S1. Primer sequences for qRT-PCR analyses. We designed all the primers using https://sg.idtdna.com/pages/products/custom-dna-rna combine with the NCBI and UCSC websites. Table S2. Chemical characteristics of the PM samples used for functional analyses. Water soluble ions were analyzed by ion chromatography, element measurement by inductivity coupled plasma-mass spectrometry (ICP-MS); and organic carbon (OC) and elemental carbon (EC) fractions by DRI model 2001 carbon analyzer. Figure S1. The average concentration of PM2.5 and PM10 in the three Chinese cities (Beijing, Taiyuan, Shijiazhuang) during the study period. We collected environmental data of 3 weeks preceding the sampling. The actual time period of sampling in the three cities: 10/04/2014–26/04/2014 in Shijiazhuang, Taiyuan: 21/02/2014–01/05/2014 and Beijing: 26/09/2014–16/10/2014. Figure S2. Size distribution and protease activity of the PM sample. A. 20 × microscopic view of PM suspension showing the distribution of PM2.5 and PM10; B. protease activity of the PM samples based on FITC-labeled casein cleavage hydrolysis assay. The protease activities are measured by mean fluorescence level with standard deviation. Figure S3. Expression levels of T-cell related cytokines in mouse serum follow the treatment of PM. Figure S4. Differentiation statuses of T-cell subtypes in mouse lung following the treatment of PM suspension. T-cell differentiation is evaluated by flow cytometry at 18 h, 24 h, 40 h and 72 h after the treatment. [file 12931_2020_1368_MOESM1_ESM.zip › revision_figure_S3.pdf]

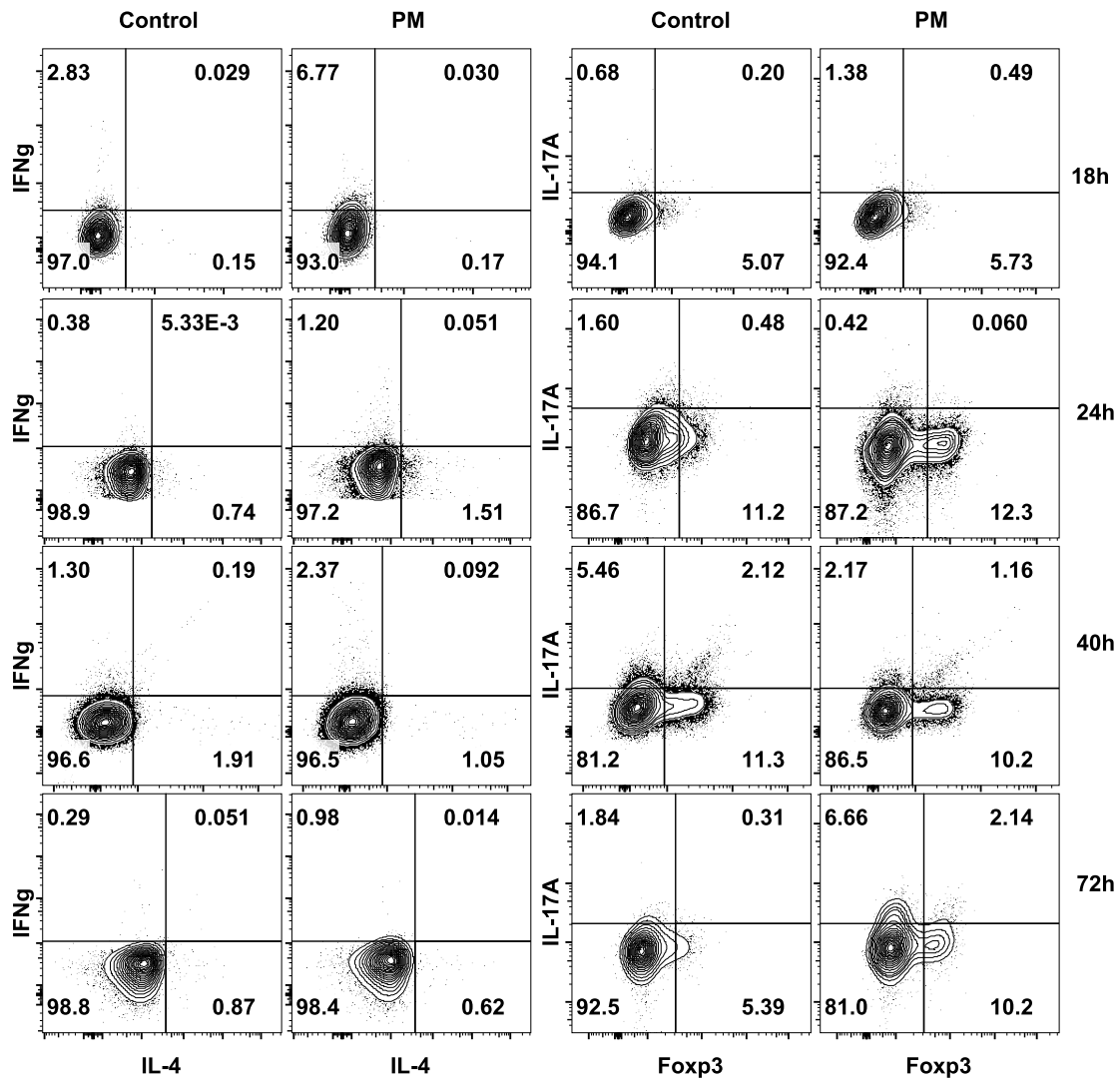

Supplement: Supplementary file 1 — Additional file 1: Reagents and Antibodies. Table S1. Primer sequences for qRT-PCR analyses. We designed all the primers using https://sg.idtdna.com/pages/products/custom-dna-rna combine with the NCBI and UCSC websites. Table S2. Chemical characteristics of the PM samples used for functional analyses. Water soluble ions were analyzed by ion chromatography, element measurement by inductivity coupled plasma-mass spectrometry (ICP-MS); and organic carbon (OC) and elemental carbon (EC) fractions by DRI model 2001 carbon analyzer. Figure S1. The average concentration of PM2.5 and PM10 in the three Chinese cities (Beijing, Taiyuan, Shijiazhuang) during the study period. We collected environmental data of 3 weeks preceding the sampling. The actual time period of sampling in the three cities: 10/04/2014–26/04/2014 in Shijiazhuang, Taiyuan: 21/02/2014–01/05/2014 and Beijing: 26/09/2014–16/10/2014. Figure S2. Size distribution and protease activity of the PM sample. A. 20 × microscopic view of PM suspension showing the distribution of PM2.5 and PM10; B. protease activity of the PM samples based on FITC-labeled casein cleavage hydrolysis assay. The protease activities are measured by mean fluorescence level with standard deviation. Figure S3. Expression levels of T-cell related cytokines in mouse serum follow the treatment of PM. Figure S4. Differentiation statuses of T-cell subtypes in mouse lung following the treatment of PM suspension. T-cell differentiation is evaluated by flow cytometry at 18 h, 24 h, 40 h and 72 h after the treatment. [file 12931_2020_1368_MOESM1_ESM.zip › revision_figure_S4.pdf]
